# Molecular Characterization and Prognosis of Lactate-Related Genes in Lung Adenocarcinoma

**DOI:** 10.3390/curroncol30030217

**Published:** 2023-02-27

**Authors:** Zixin Guo, Liwen Hu, Qingwen Wang, Yujin Wang, Xiao-Ping Liu, Chen Chen, Sheng Li, Weidong Hu

**Affiliations:** 1 Department of Thoracic Surgery, Zhongnan Hospital of Wuhan University, Wuhan 430071, China; 2 Department of Biological Repositories, Zhongnan Hospital of Wuhan University, Wuhan 430071, China; 3 Hubei Key Laboratory of Tumor Biological Behaviors & Hubei Cancer Clinical Study Center, Wuhan 430071, China; 4 Department of Urology, Zhongnan Hospital of Wuhan University, Wuhan 430071, China; 5 Human Genetics Resource Preservation Center of Hubei Province, Wuhan 430071, China

**Keywords:** lactate, lung adenocarcinoma, immune infiltration, prognosis, tumor microenvironment

## Abstract

Objective: To explore the lactate-related genes (LRGs) in lung adenocarcinoma (LUAD) by various methods, construct a prognostic model, and explore the relationship between lactate subtypes and the immune tumor microenvironment (TME). Methods: 24 LRGs were collected. The mutation landscape and the prognosis value of LRGs were explored by using The Cancer Genome Atlas (TCGA) data. Consensus clustering analysis was used for different lactate subtype identification. Based on the lactate subtypes, we explore the landscape of TME cell infiltration. A risk-score was calculated by using the LASSO-Cox analysis. A quantitative real-time PCR assay was utilized to validate the expression of characteristic genes in clinical cancer tissues and paracarinoma tissues from LUAD patients. Results: Comparing the normal samples, 18 LRGs were differentially expressed in tumor samples, which revealed that the differential expression of LRGs may be related to Copy Number Variation (CNV) alterations. The two distinct lactate subtypes were defined. Compared to patients in the LRGcluster A group, LUAD patients in the LRGcluster B group achieved better survival. The prognostic model was constructed based on differentially expressed genes (DEGs) via the LASSO-Cox analysis, which showed the accuracy of predicting the prognosis of LUAD patients using the ROC curve. A high-risk score was related to a high immune score, stromal score, and tumor mutation burden (TMB). Patients had better OS with low risk compared with those with high risk. The sensitivities of different risk groups to chemotherapeutic drugs were explored. Finally, the expression of characteristic genes in clinical cancer tissues and paracarinoma tissues from LUAD patients was verified via qRT-PCR. Conclusions: The lactate subtypes were independent prognostic biomarkers in LUAD. Additionally, the difference in the lactate subtypes was an indispensable feature for the individual TME. The comprehensive evaluation of the lactate subtypes in the single tumor would help us to understand the infiltration characteristics of TME and guide immunotherapy strategies.

## 1. Introduction

Lung cancer is one of the most common malignant tumors that are harmful to human health worldwide. Its incidence rate and mortality rate are the highest [1]. Lung adenocarcinoma (LUAD) is the most common pathological subtype of non-small cell lung cancer (NSCLC). The incidence rate of LUAD has increased year by year. Despite the progress in cancer treatment, the 5-year overall survival rate of LUAD still lingers around 20% [2]. The initial symptoms of LUAD are not obvious and are often easily overlooked. Patients with LUAD were already at an advanced stage when disease symptoms were apparent. Molecular-targeted therapy and immune checkpoint inhibitor therapy have exhibited powerful efficacy in patients with LUAD in recent years [3,4]. However, the problem of treatment insensitivity and resistance remains for a large proportion of patients [5]. Therefore, the research of LUAD still needs to be continuously pioneered.

Even when oxygen is sufficient to support mitochondrial oxidative phosphorylation, tumor tissues tend to convert glucose to lactate, which is a metabolic hallmark of aerobic glycolysis, and is called the Warburg effect. It is mainly characterized by high levels of metabolite lactate, the rapid depletion of glucose, and active glycolysis [6,7]. The large amount of lactate generated by glycolysis in the tumor microenvironment (TME) has previously been considered merely a metabolic waste. However, more and more studies have provided evidence that lactate can play a role in promoting neoplastic processes. Lactate, as an immunosuppressive molecule, can promote the immune escape of tumors. Lactate can activate the ERK/STAT3 signaling pathway to promote the polarization of tumor-associated macrophages (TAMs) toward the M2 phenotype [8]; in response to the M2 secreted anti-inflammatory and proangiogenic cytokines, tumors tend to survive, grow, and metastasize [9]. The high levels of lactate dehydrogenase (LDH) tend to be related to poor overall survival as well as progression-free survival in metastatic prostate cancer [10]. Therefore, exploring the mechanism of lactate-related genes (LRGs) in the TME of LUAD could help deepen our understanding of LUAD.

The RNA sequencing and clinicopathological data of LUAD were downloaded from The Cancer Genome Atlas (TCGA). Patients with LUAD were classified into two distinct subtypes according to LRGs and divided into three gene subtypes according to the differentially expressed genes (DEGs) between the lactate subtypes. We found that the TME characteristics in these subtypes were highly correlated with the prognosis of LUAD patients and the relative abundance of infiltrating cells. Finally, a scoring system was developed to predict the prognosis, which could help to understand the potential mechanism of lactate on the development of LUAD.

## 2. Materials and Methods

### 2.1. Data Resources

The transcriptome, simple nucleotide variation (SNV) and clinicopathological data of LUAD were downloaded from TCGA database (https://portal.gdc.cancer.gov/, accessed on 19 April 2022), containing 535 LUAD samples and 59 normal samples. The transcriptome data format was FPKM and was subsequently converted to TPM. The clinical information included survival information, gender, age, TNM, and stage. The Copy Number Variation (CNV) data of TCGA-LUAD were downloaded from the UCSC Xena (http://xena.ucsc.edu/, accessed on 19 April 2022). GSE68465 [11] was downloaded from the GEO database (https://www.ncbi.nlm.nih.gov/geo/, accessed on 19 April 2022), which contains 443 LUAD samples with the gene expression and follow up data of the tumor metastasis. TCGA-LUAD and GSE68465 were standardized and integrated together via the “sva” package.

### 2.2. Mutation, CNV, Expression, and Survival of LRGs in LUAD

24 LRGs were retrieved from the MSigDB database [12,13], including the “GOBP lactate metabolic process”, “GOBP lactate transmembrane transport”, “GOMF lactate dehydrogenase activity”, and the “GOMF lactate transmembrane transporter activity”, which was listed in Table S1. The waterfall plot of the LRGs in LUAD was performed using the “maftools” [14] package according to the SNV data. Somatic copy number alterations in these LRGs were explored using the CNV data. The expression of LRGs in LUAD and normal tissues was analyzed using the Wilcoxon test. The Kaplan–Meier method and log-rank test were used for survival analysis of LRGs using the “survival” package based on the TCGA-LUAD data.

### 2.3. Consensus Clustering for LRGs

Cluster analysis was performed using the R package “ConsensusClusterPlus” [15], using agglomerative km clustering with a euclidean distances and by resampling 80% of the samples for 1000 repetitions. The average consistency evaluation within the cluster group was used to determine the optimal number of clusters. For the prognostic comparison of clusters, the Kaplan–Meier method was used via R package “survival”. We followed the methods of Shao et al. [16] to investigate the gene function of the LRGs using Gene set variation analysis (GSVA) and single-sample gene set enrichment analysis (ssGSEA) [17]. “c2.cp.kegg.v6.2.symbols.gmt” was chosen as the enrichment gene set. Gene set enrichment analysis (GSEA) (http://software.broadinstitute.org/gsea/index.jsp, accessed on 19 April 2022) [12] was conducted between the LRGcluster A and LRGcluster B. In this study, nominal *p* < 0.05, and FDR < 25% were considered significantly enriched.

### 2.4. LRGs-Related Genes Identification and Function

The “limma” package was used to analyze the DEGs between the two subtypes of LRGs. Adjusted *p* < 0.05 and Log2|FC| > 1.5 was set as the filtering criteria for DEGs. The DEGs were regarded as LRGs-related genes.

### 2.5. Development and Validation of the Prognostic Model

Firstly, genes associated with prognosis were analyzed using univariate Cox regression analysis based on the LRGs-related genes via the R package “survival”. A LRGs-related gene with *p* < 0.05 was related to the prognosis. Secondly, patients with LUAD were classified into several gene clusters via consensus clustering analysis, as we previously did. The survival rate of the several gene clusters was compared using the Kaplan–Meier method via the “survival” package. The expression levels of LRGs between the gene clusters were compared using the Wilcoxon test. In order to increase the reliability of the results, the samples with LUAD were divided 1:1 into training groups (*n* = 471) and testing groups (*n* = 471). To prevent overfitting of the prognostic model, we included LRGs-related genes that related to prognosis and to develop the model using the LASSO-Cox analysis. There were three genes, which were used to construct the model using the penalty parameter (λ). After defining the risk score, the risk score differences between different LRGclusters and different gene clusters were compared using the Wilcoxon test. An alluvial diagram was drawn to show the changes in the LRGclusters, gene clusters, survival status, and risk score by using R package “ggplot2”. In addition, the survival in the training and testing sets was determined used the Kaplan–Meier method. The predictive ability of the prognostic model was tested using the receiver operating curves (ROC) of 1, 3, and 5 years using the R package “survivalROC”.

### 2.6. Development and Validation of the Nomogram

The predictive nomogram was established based on the risk score and clinical characteristics using the “rms” package [18]. Calibration plots were used to assess the performance of the nomogram by comparing the nomogram-predicted survival with the actual observed outcome.

### 2.7. Landscape of TME Cell Infiltration between Low- and High-Risk Groups

The ESTIMATE was used to evaluate the stromal and immune scores of each sample according to the TCGA-LUAD dataset [19]. In addition, the CIBERSORT was used to calculate the fractions of 22 human immune cell of each sample [20]. We followed the methods of Shao et al. [16] to investigate the abundance of immune cells in different groups using the Wilcoxon test and Spearman’s correlation analysis. Differences in the immune checkpoints’ expression between the high- and low-risk groups were also analyzed, including 24 inhibitory immune checkpoints and 36 stimulatory immune checkpoints, via the Wilcoxon test [21]. Furthermore, we analyzed the relationships between the cancer stem cell (CSC) and the two risk groups by spearman correlation analysis.

### 2.8. Mutation and Drug Susceptibility Analysis

Based on the SNV data of TCGA-LUAD, the somatic mutations of LUAD patients between the two groups was performed using the “maftools” R package. Then, the differences in the tumor mutation burden (TMB) were analyzed busing the Wilcoxon test and Spearman’s correlation analysis. The semi-inhibitory concentration (IC50) values were calculated using the package “pRRophetic” [22] to explore the differences between the two groups using the Wilcoxon test. The pRRophetic algorithm constructed ridge regression models to predict drug IC50 based on the GDSC (Genomics of Drug Sensitivity in Cancer) cell line expression profiles and TCGA gene expression profiles.

### 2.9. Quantitative Real-Time PCR (qRT-PCR) Assay

We collected 10 cancer tissues and 10 paracarinoma tissues from LUAD patients who underwent radical pneumonectomy in Zhongnan Hospital of Wuhan University. All patients were pathologically diagnosed with lung adenocarcinoma. Paracarinoma tissues were defined as that taken approximately 2.5 cm from the lesion. After collecting the samples, we assigned them individual numbers and then immediately placed them in liquid nitrogen tanks for preservation. Informed consent was obtained from all participating individuals, and the study was approved by the Ethics Committee at Zhongnan Hospital of Wuhan University. To further analyze the roles of genes in LUAD, total RNAs from the 20 samples were extracted via an RNA Isolation Kit (Vazyme Biotech Co., Ltd, Nanjing, China) according to the manufacturer’s protocol. Reverse transcription was performed based on the HiScript RT SuperMix (Vazyme Biotech Co., Ltd, Nanjing, China). Quantitative real-time PCR was performed using the CFX96 Real-Time System (Bio-Rad, Hercules, California, USA). The transcription level of GAPDH was used as an internal control. Relative gene expression levels were determined using the 2-ΔΔCt method. The differences in gene expression between cancer tissues and paracarinoma tissues were analyzed using the Mann–Whitney U test. The PCR primers were as follows: CACNA2D gene 5′-CCTGATGCTGGCACTCTACAATA-3′ (Forward) and 5′-TGGTAGATGAGGCCGTAGAGAAG-3′ (Reverse); CYP2B7P gene 5′-CCCCTTTTGGGGAACCTTCT-3′ (Forward) and 5′-GACTGGGTCCATGATGACGAT-3′ (Reverse); KRT6A gene 5′-AACTTCCTGAGAGCCTTGTATGA-3′ (Forward) and 5′-ATCTCCTCATATTGGGCCTTGAC-3′ (Reverse); GAPDH gene 5′-CAGAACATCATCCCTGCCTCTAC-3′ (Forward) and 5′-ATGAAGTCAGAGGAGACCACCTG-3′ (Reverse).

### 2.10. Statistical Analysis

The Kaplan–Meier method and log-rank test were used for survival analysis. Statistical significance was set at *p* < 0.05. All statistical analyses were performed using R software (v4.1.0) (Robert Gentleman and Ross Ihaka, Auckland, New Zealand).

## 3. Results

### 3.1. Mutation, CNV, Expression, and Survival of LRGs in LUAD

**Figure 1:**
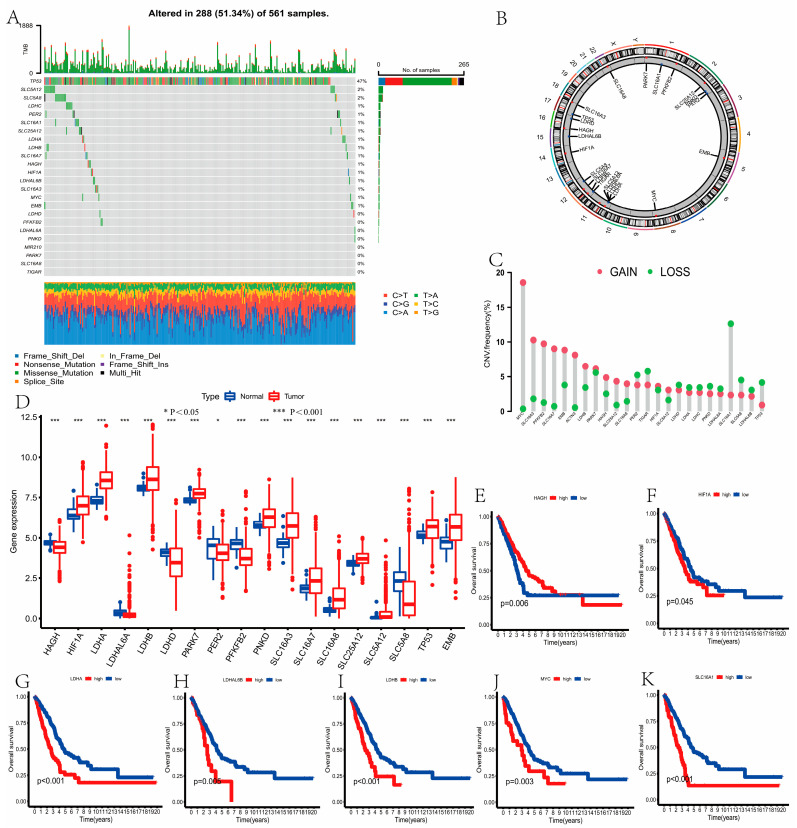
Mutation, CNV, expression and survival of lactate-related genes (LRGs) in lung adenocarcinoma (LUAD). (**A**) The mutation of LRGs in LUAD. (**B**) The locations of the CNV alterations in the LRGs. (**C**) The CNV alteration frequency of LRGs in LUAD. (**D**) The expression of LRGs between LUAD and normal samples. Comparing the normal samples, 18 LRGs were differentially expressed in tumor samples using the Wilcoxon test. (**E**–**K**) The high expression groups of HAGH had a better prognosis. The low expression groups of HIF1A, LDHA, LDHAL6B, LDHB, MYC, and SLC16A1 had a better prognosis using Kaplan–Meier method and log-rank test (*p* < 0.05). * *P* < 0.05, *** *P* < 0.001.

**Figure 2:**
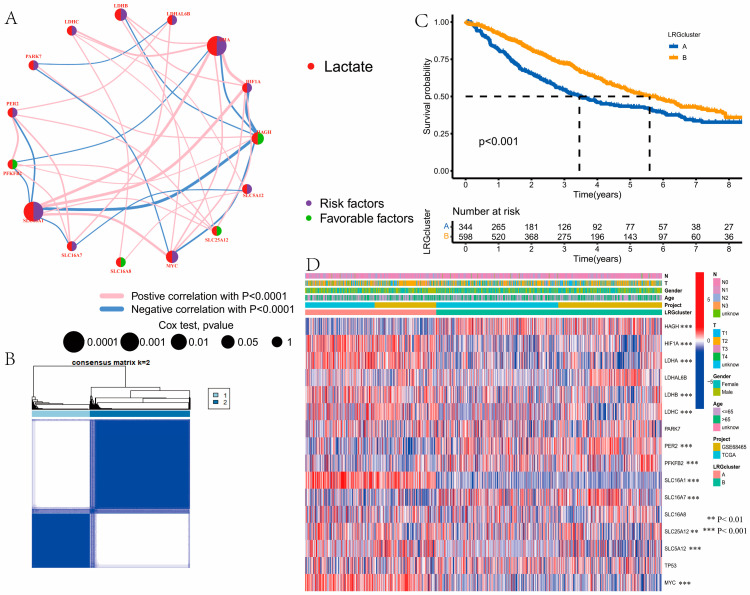
Identification of lactate clusters in lung adenocarcinoma (LUAD). (**A**) The lactate-related gene (LRG) interactions and their prognostic value in LUAD. (**B**) The result of consensus clustering analysis based on LRGs. The samples with LUAD were classified into LRG cluster A (*n* = 344) and B (*n* = 598), which were the best choices when *k* = 2. (**C**) Differences in overall survival (OS) between the two distinct subtypes. The patients in LRG cluster B had a better prognosis than patients in LRG cluster A (log-rank test, *p* < 0.001). (**D**) The expression levels of LRGs and clinicopathological features of samples with LUAD between the two LRG clusters. ** *P* < 0.01, *** *P* < 0.001.

The detailed flow chart of the study is displayed in Figure S1. In total, 24 LRGs were included in this study (Table S1). Based on the SNV data, we found that the mutation frequency of genes in LUAD was very low, except for TP53, which had a mutation frequency of 47% in 561 samples with LUAD (Figure 1A). Figure 1B showed the locations of the CNV alterations in the LRGs on their respective chromosomes. Somatic copy number alterations of the LRGs were explored, and we discovered that copy number alterations were present in some LRGs. Among them, MYC, SLC16A3, PFKFB2, SLC16A7, EMB, ACTN3, and LDHB had widespread CNV increases, while SLC16A1, TIGAR, PER2, and PARK7 showed CNV decreases (Figure 1C). Comparing the normal samples, 18 LRGs were differentially expressed in tumor samples, which revealed that the differential expression of LRGs may be related to CNV alterations (*p* < 0.05, Figure 1D). Next, we concluded that the expression of seven PRGs was related to the prognosis in the patients with LUAD by using the Kaplan–Meier method. Among them, the high expression groups of HAGH had a better prognosis (*p* < 0.05, Figure 1E), and the high expression group of HIF1A, LDHA, LDHAL6B, LDHB, MYC, and SLC16A1 had a worse prognosis based on the results of the survival curve (*p* < 0.05, Figure 1F–K). Figure 2A showed the LRGs interactions and their prognostic value in LUAD.

### 3.2. Identification of Lactate Clusters in LUAD

Cluster analysis was used to classify the samples with LUAD to explore the LRGs based on the expression of 16 PRGs, which were intersected in TCGA-LUAD and GSE68465. The samples with LUAD were classified into LRG cluster A (*n* = 344) and B (*n* = 598), which were the best choices when k = 2(Figure 2B). We concluded that patients in LRG cluster B had a better prognosis than patients in LRG cluster A (log-rank test, *p* < 0.001; Figure 2C). Then, we drew the heatmap to show the expression levels of LRGs and clinicopathological features of samples with LUAD between the two LRG clusters (Figure 2D).

### 3.3. Gene Function Analysis for LRGs

**Figure 3:**
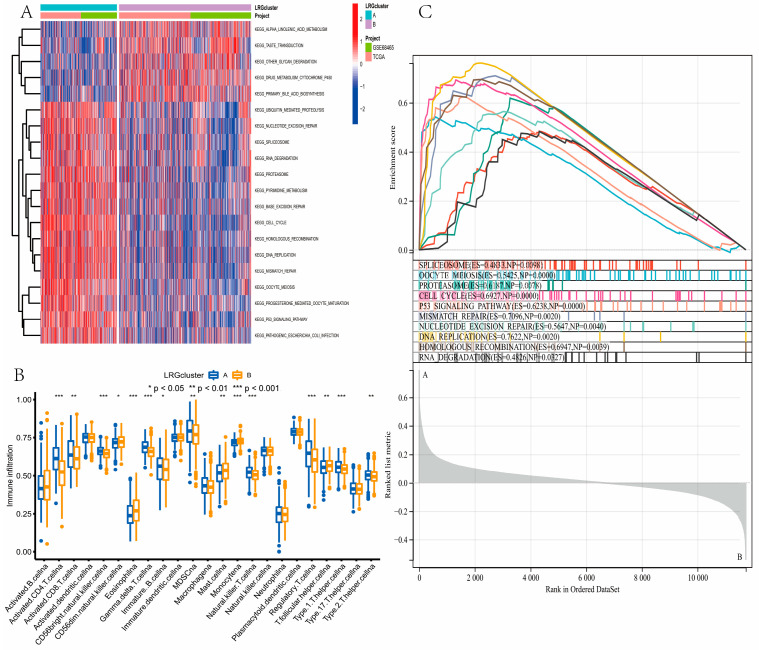
Correlations of tumor microenvironment (TME) and two lung adenocarcinoma (LUAD) subtypes. (**A**) GSVA of biological pathways between two distinct subtypes. LRG cluster B was enriched in alpha linolenic acid metabolism, taste transduction, other glycan degradation, drug metabolism cytochrome P450, and primary bile acid biosynthesis pathways. (**B**) Abundance of 22 infiltrating immune cell types in the two LRG clusters. Most immune cell infiltrates were significantly different between the two LRG clusters. (**C**) The results of the GSEA between LRG cluster A and LRG cluster B. * *P* < 0.05, ** *P* < 0.01, *** *P* < 0.001.

GSVA analysis revealed that LRG cluster B was enriched in alpha linolenic acid metabolism, taste transduction, other glycan degradation, drug metabolism cytochrome P450, and primary bile acid biosynthesis pathways (Figure 3A; Table S2). ssGSEA was performed to analyze the differences in immune infiltrating cells among different subtypes. Most immune cell infiltrates were significantly different between the two LRG clusters (Figure 3B). The GSEA showed that LRG cluster A was enriched in the spliceosome, oocyte meiosis, proteasome, cell cycle, P53 signaling pathway, mismatch repair, nucleotide excision repair, DNA replication, homologous recombination, and RNA degradation (Figure 3C).

### 3.4. Identification of Gene Clusters

**Figure 4:**
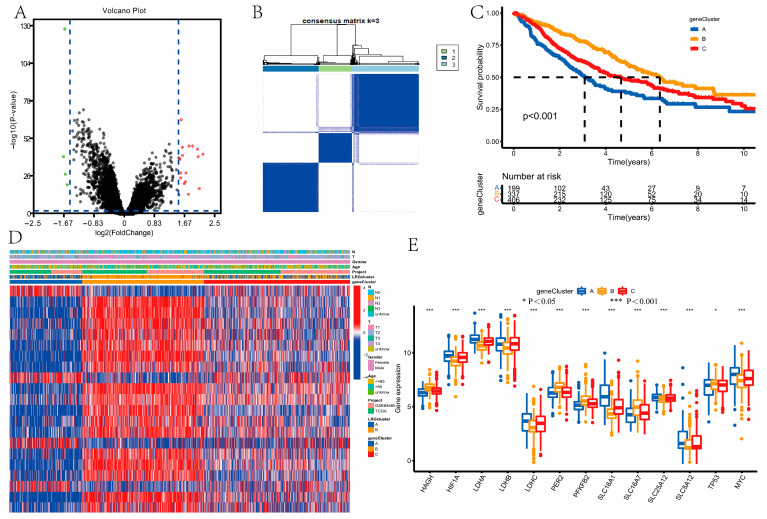
Identification of gene subtypes. (**A**) Differentially expressed genes (DEGs) between the two LRGclusters. (**B**) The result of consensus clustering analysis based on DEGs. (**C**) Differences in overall survival between the distinct gene subtypes. The patients in gene cluster B had the best prognosis, whereas patients in gene cluster A had the worst prognosis, using the Kaplan–Meier method and log-rank test (*p* < 0.001). (**D**) The expression levels of lactate-related genes (LRGs) and clinicopathological features of samples with lung adenocarcinoma (LUAD) between the three gene clusters. (**E**) Differences in the expression of LRGs. The three gene clusters showed significant differences in LRGs expression using Wilcoxon test. * *P* < 0.05, *** *P* < 0.001.

21 DEGs were identified via the package “limma,” and included in functional enrichment analysis (Figure 4A). We then screened out all genes related to OS time via a univariate Cox regression analysis (*p* < 0.05, Table S3). Based on 21 prognostic genes, the LUAD samples were classified into gene clusters A, B, and C via cluster analysis (Figure 4B). We concluded that patients in gene cluster B had the best prognosis, whereas patients in gene cluster A had the worst prognosis (*p* < 0.001; Figure 4C). Furthermore, we drew the heatmap to show the expression levels of prognostic genes and clinicopathological features of samples with LUAD among these three gene clusters (Figure 4D). The three gene clusters showed significant differences in LRGs expression (Figure 4E).

### 3.5. Construction and Validation of the Prognostic Model

**Figure 5:**
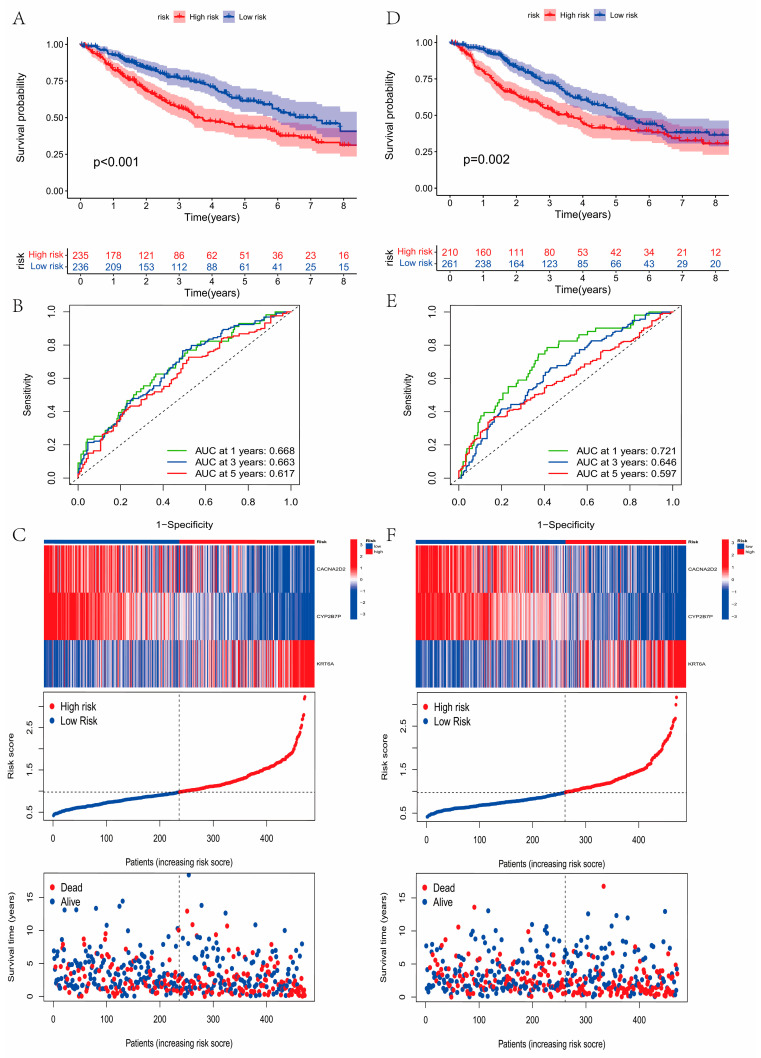
Construction and validation of the prognostic model. The differences in overall survival (OS) between high- and low-risk groups. The patients in low-risk group had significantly higher OS, using the Kaplan–Meier method and log-rank test ((**A**), training group; (**D**), testing group). ROC curve measuring the predictive value of the risk score. The model showed better accuracy in predicting survival ((**B**), training group; (**E**), testing group). The heatmap of the prognostic model ((**C**), training group; (**F**), testing group).

**Figure 6:**
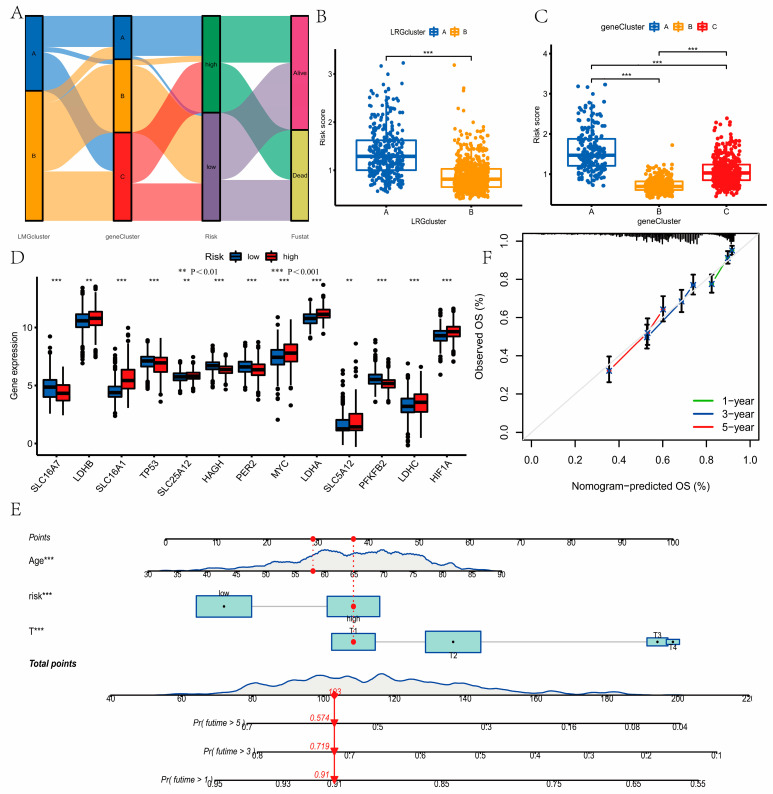
(**A**) The changes in patients with lung adenocarcinoma (LUAD) in the LRG clusters, gene clusters, survival status, and risk score. (**B**) The differences in the risk score between LRG cluster A and LRG cluster B. Compared with LRG cluster A, LRG cluster B had lower risk score, using the Wilcoxon test. (**C**) The differences in the risk score among gene clusters A, B and C. Gene cluster B and gene cluster C had lower risk scores compared with gene cluster A, using the Wilcoxon test. (**D**) Differences in the expression of lactate-related genes (LRGs) of the two risk groups. The low- and high-risk groups showed significant differences in LRGs expression using Wilcoxon test. (**E**) Nomogram for predicting the overall survival (OS) of LUAD. (**F**) Calibration curves of the nomogram. Compared to an ideal model, the nomogram had a better performance. ** *P* < 0.01, *** *P* < 0.001.

Firstly, the patients with LUAD were randomly classified into training (*n* = 471) and testing (*n* = 471) groups using package “caret”. Then we included the 21 prognostic DEGs into the LASSO-Cox analysis to establish the model. Three genes, CACNA2D2, CYP2B7P, and KRT6A, were selected to establish the model according to the minimal criterion. The following is the formula:

Risk score = (−0.0652) ∗ CACNA2D2 + (−0.1039) ∗ CYP2B7P + (0.0704) ∗ KRT6A,

Based on the median risk score, the samples with LUAD were classified into low- and high-risk groups. Figure S2 showed the expression difference in these three genes in the low-risk and high-risk groups. We concluded that patients in the low-risk group had significantly higher OS (*p* < 0.001, Figure 5A,D). The heatmap of the prognostic model is shown in Figure 5C,F. Here, the model showed a better accuracy in predicting survival at 1 year (AUC = 0.668), 3 years (AUC = 0.663), and 5 years (AUC = 0.617, Figure 5B) in training groups, and 1 year (AUC = 0.721), 3 years (AUC = 0.646), and 5 years (AUC = 0.597, Figure 5E) in testing groups. Figure S2 showed that CACNA2D2 and CYP2B7P were highly expressed in the low-risk groups, and KRT6A was highly expressed in the high-risk groups. These results were consistent in the training and testing groups, indicating the reliability and stability of the above results. Figure 6A showed the changes in patients with LUAD in the LRG clusters, gene clusters, survival status, and risk score. Compared with LRG cluster A and gene cluster A, LRG cluster B, gene cluster B, and gene cluster C had lower risk scores (*p <* 0.001, Figure 6B,C). The low- and high-risk groups showed significant differences in LRG expression (Figure 6D).

### 3.6. Development of a Nomogram to Predict Survival

In order to more conveniently predict 1-, 3-, and 5-year OS, we incorporated risk score, age, and T stage to establish the nomogram (Figure 6E). Compared to an ideal model, the nomogram had a better performance through the calibration plots (Figure 6F).

### 3.7. Evaluation of TME Cell Infiltration between the High- and Low-Risk Groups

**Figure 7:**
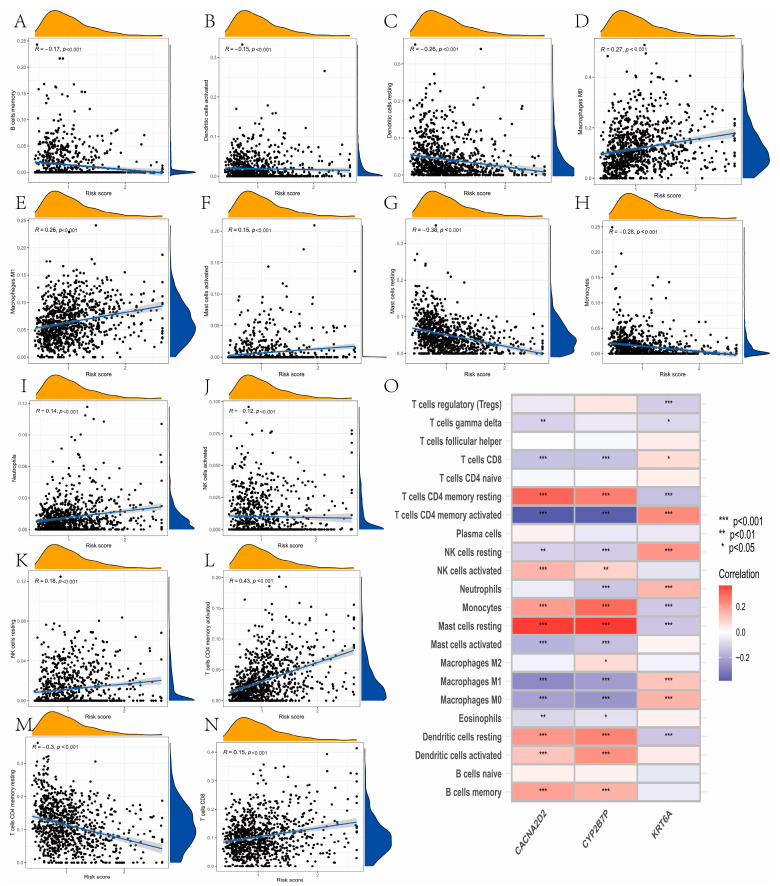
Evaluation of cell infiltration between the high- and low-risk groups. (**A**–**N**) Correlations between immune cell types and risk score using Spearman’s correlation analysis. The risk score was negatively correlated with memory resting T cells CD4, activated NK cells, monocytes, resting mast cells, resting dendritic cells, activated dendritic cells, and B cells’ memory. Additionally, the risk score was positively associated with T cells CD8, memory activated T cells CD4, resting NK cells, neutrophils, activated mast cells, macrophage M1, and macrophage M0. (**O**) These three genes selected in the prognostic model were significantly related to most immune cells using Spearman’s correlation analysis. * *P* < 0.05, ** *P* < 0.01, *** *P* < 0.001.

**Figure 8:**
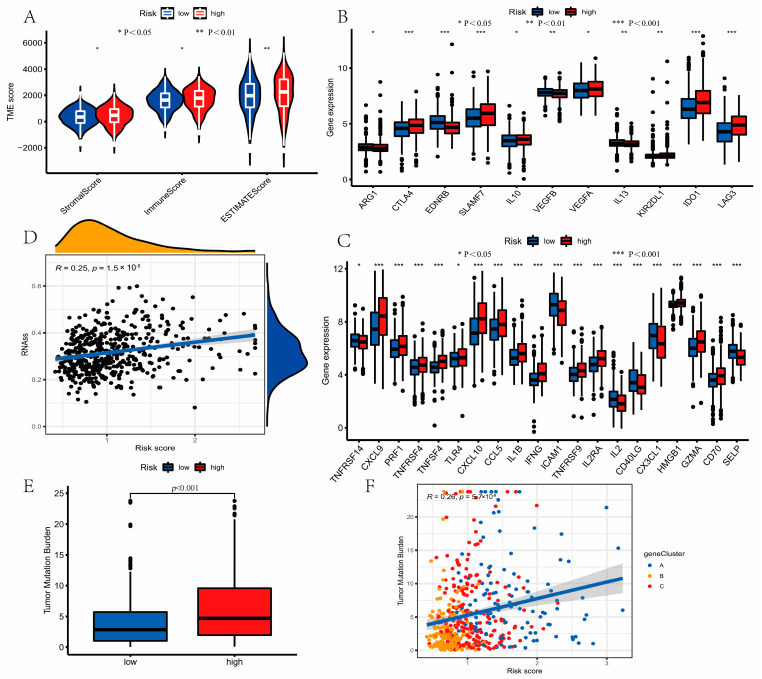
Evaluation of tumor microenvironment (TME) and tumor mutation burden (TMB) between the high- and low-risk groups. (**A**) The differences in stromal score and immune score between risk groups. A high-risk score was closely related to a high stromal score and immune score. (**B**,**C**) The expression of 11 inhibitory immune checkpoints and 20 stimulatory immune checkpoints was significantly different between the distinct risk groups, using the Wilcoxon test. (**D**) The correlations between the CSC index and risk score using Spearman’s correlation analysis. (**E**,**F**) The correlations between TMB and risk score using Spearman’s correlation analysis. Compared with the high-risk group, the low-risk group had lower TMB. * *P* < 0.05, ** *P* < 0.01, *** *P* < 0.001.

The risk score was negatively correlated with resting memory T cells CD4, activated NK cells, monocytes, resting mast cells, resting dendritic cells, activated dendritic cells, and B cells memory via the CIBERSIRT algorithm. Additionally, the risk score was positive associated with T cells CD8, memory activated T cells CD4, resting NK cells, neutrophils, activated mast cells, macrophage M1, and macrophage M0 (Figure 7A–N). We also concluded that these three genes selected in the prognostic model were significantly related to most immune cells (Figure 7O). A high-risk score was closely related to a high stromal score and immune score, whereas a low-risk score was related to a low immune score and stromal score (Figure 8A). The expression of 11 inhibitory immune checkpoints and 20 stimulatory immune checkpoints was significantly different between distinct risk groups (Figure 8B,C). Furthermore, a high CSC index was related to a high-risk score (R = 0.25, *p* < 0.001), and we concluded that samples with high risk had a lower degree of cell differentiation (Figure 8D).

### 3.8. Mutation and Drug Susceptibility Analysis

**Figure 9:**
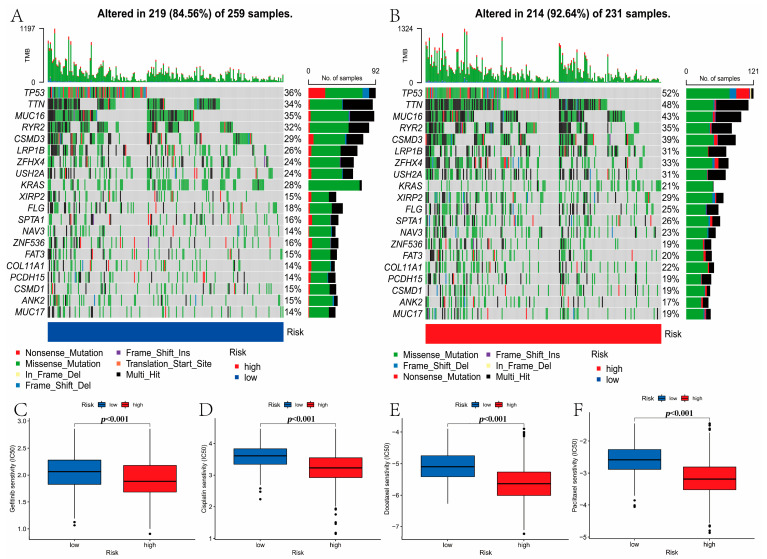
Mutation and drug susceptibility analysis. The waterfall plot of somatic mutation features established with low-risk groups (**A**) and high-risk groups (**B**). The frequency of gene mutation was lower in the low-risk group. (**C**–**F**) The patients in the high-risk group had lower IC50 value in these drugs: (**C**) Gefitinib, (**D**) Cisplatin, (**E**) Docetaxel, and (**F**) Paclitaxel.

We concluded that compared with the high-risk group, the low-risk group had lower TMB (*p* < 0.001, Figure 8E,F). Next, the differences in the somatic mutations between the two risk groups were analyzed based on the TCGA-LUAD. The frequency of gene mutation was lower in the low-risk group, which was consistent with that of TMB (Figure 9A,B). Finally, we concluded that the patients in the high-risk group had lower IC50 value for Gefitinib, Cisplatin, Docetaxel, and Paclitaxel (Figure 9C–F). These results revealed that LRGs might be related to drug sensitivity.

### 3.9. Quantitative Real-Time PCR Validation

**Figure 10:**
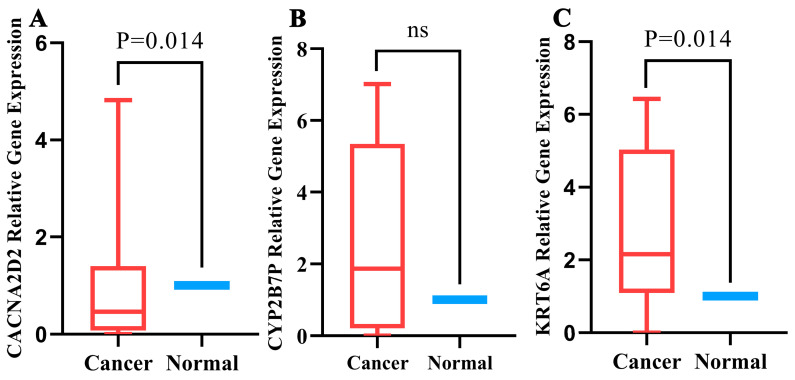
The relative expression levels of the 3 genes in normal and lung adenocarcinoma (LUAD) tissues using qRT-PCR. The expression levels of CACNA2D2 were downregulated in LUAD tissues (**A**) There was a trend of upregulation of CYP2B7P in LUAD tissues (**B**) and that KRT6A was upregulated in LUAD tissues (**C**).

To verify the expression levels of CACNA2D2, CYP2B7P, and KRT6A in LUAD tissues, quantitative real-time PCR validation was performed. We found that the expression levels of CACNA2D2 were downregulated in LUAD tissues (Figure 10A) and that KRT6A was upregulated in LUAD tissues, compared with paracarinoma tissues (Figure 10C). There was a trend of upregulation of CYP2B7P in LUAD tissues, but it was not statistically significant and may be correlated with the sample size (Figure 10B). Therefore, we speculate that CACNA2D2, CYP2B7P, and KRT6A may be reliable biomarkers for LUAD.

## 4. Discussion

Glycolysis is a major way for cancer cells to obtain energy under hypoxic conditions, but upregulation of glycolysis is accompanied by the production of large amounts of acidic substances such as lactate and pyruvate, reducing the intracellular PH value. When acidic products accumulate in excess, they can lead to the acidification of the intracellular environment, causing its growth inhibition and even tumor cell apoptosis [23]. In order to maintain PH homeostasis, tumor cells can expel intracellular lactate and hydrogen ions via transport receptors while acidifying the extracellular environment [24]. Although lactic acid fermentation is less efficient than oxidative phosphorylation, it offers tumor cells several competitive advantages, containing the acidification of the TME, the application of intermediates to multiple biosynthetic pathways, and rapid ATP production. Lactate is a major component in maintaining the acidic phenotype of the TME and contributes to some important biological features of tumors, including cell migration, invasion, and angiogenesis, among others [23]. Transmembrane transport of lactate depends on monocarboxylate transporters (MCT), encoded by the solute carrier (SLC) 16 gene family, of which MCT1 (SLC16A1), MCT2 (SLC16A7), MCT3 (SLC16A8), and MCT4 (SLC16A3) were demonstrated to be capable of the bidirectional transmembrane transport of pyruvate and lactate [25]. Upregulation of MCT1/4 has been found in tumor cells of several tumors, including breast, lung, kidney, and melanoma [26,27], and may be associated with the regulation of hypoxia, the P53 and MYC genes, ERK-MAPK/AMPK signaling pathway, and so on. As important lactate transporters, MCT1 and MCT4 play an integral role in metabolic adaptation, metabolic symbiosis, and maintenance of the metabolic phenotype of tumor cells [28,29,30,31]. Given the effect on tumor cells, MCT1/4 has been suggested to be a marker of shorter survival in several tumors containing head and neck, melanoma, lung, and breast cancers [32,33], and is related to poor prognosis and a later tumor stage in cancer patients [34]. Studies have shown that high GLUT1 (glucose transporter type 1) and high MCT4 expression are related to worse disease-specific survival in adenocarcinoma (*p* = 0.032) [35].

More and more evidence showed that lactate played an important role in the occurrence and development of tumors. In this study, we firstly collected 24 lactate-related genes from the MSigDB database. We discovered that copy number alterations were present in some LRGs. Among them, MYC, SLC16A3, PFKFB2, SLC16A7, EMB, ACTN3, and LDHB had widespread CNV increases, while SLC16A1, TIGAR, PER2, and PARK7 showed CNV decreases. Comparing the normal samples, 18 LRGs were differentially expressed in tumor samples, which revealed that the differential expression of LRGs may be related to CNV alterations. These LRGs might work with each other, suggested by the correlation analysis and PPI network. Then based on the 16 LRGs, the two distinct clusters were immediately identified. Compared to patients in LRG cluster A group, LUAD patients in the PRG cluster B group achieved better survival. Most immune cell infiltrates were significantly different between the two LRG clusters, which demonstrated that LRGs might work in the prognosis of LUAD. Furthermore, we identified 21 LRGs-related DEGs, which were significantly enriched in lung development and immune pathways, via GO and KEGG analysis. Based on 21 genes, the LUAD patients were classified into gene clusters A, B, and C to further validate this regelation mechanism. Similarly, LUAD patients included in gene cluster B had the best prognosis, whereas patients in gene cluster A showed the worst prognosis, which indicated that lactate was of great significance in the prognosis of LUAD. Therefore, we constructed a prognostic model, which was accurate in predicting the prognostic survival. The high-risk score was closely related to a high stromal score, immune score, and TMB. High TMB has been proposed as a predictive biomarker for its response to the immune checkpoint blockade [36]. These results indicated that LRGs can be potential targets for immunotherapy of LUAD and that they deserve further exploration. Finally, we explored the sensitivity to chemotherapeutic drugs in different risk-grouping samples to provide relevant directions for the treatment of LUAD. We concluded that the patients in the high-risk group had lower IC50 value for Paclitaxel, Cisplatin, Gefitinib, and Docetaxel.

In this study, the prognosis model was established according to the expression of CACNA2D2, CYP2B7P, and KRT6A, which was accurate in predicting the OS in LUAD. Studies have shown that CACNA2D2 plays an important role in several cancers, including endometrial cancer [37], colorectal cancer [38], and NSCLC [39]. CACNA2D2 overexpression inhibited cell proliferation in NSCLC. MIR210HG could promote the tumorigenesis of NSCLC through inhibiting the CACNA2D2 [39]. CYP2B7P is downregulated in nasopharyngeal carcinoma, and its expression is lower in nasopharyngeal carcinoma tissues and cell lines. Overexpression of CYP2B7P impaired the migration and invasion abilities of nasopharyngeal carcinoma cell lines [40]. KRT6A was confirmed to be highly expressed in NSCLC, and its high expression was related to poor prognosis [41]. KRT6A functioned downstream of LSD1 and upregulated G6PD via the MYC signaling pathway and was a key driver of NSCLC progression [42]. The specific roles played by these genes in the development and progression of LUAD require further investigation.

In all, the risk score might act as a useful tool to predict the prognosis, so as to further determine the tumor immunophenotype and increase the clinical application. We confirmed that the lactate-related phenotype and risk score were closely related to the TME, and these results provided a research basis to investigate the mechanism of the development and progression of LUAD as well as to develop immunotherapy for LUAD. However, the present study still has shortcomings. We did not use our own samples for basic experimental verification and clinical verification, since we are not adequately funded to verify. However, these existing conclusions are still of research significance and deserve our further exploration.

## 5. Conclusions

In conclusion, the present study proved the lactate subtypes were independent prognostic biomarker in LUAD. Additionally, the difference in the lactate subtypes was an indispensable feature for the individual TME. The comprehensive evaluation of the lactate subtypes in single tumors would help us to understand the infiltration characteristics of TME and guide more effective immunotherapy strategies.

## Data Availability

All database generated/analyzed for this study are included/have their accession numbers included in the article.

## Supplementary Materials

The following supporting information can be downloaded at: https://www.mdpi.com/article/10.3390/curroncol30030217/s1, Figure S1: Flow diagram of this study; Figure S2: The expression difference of these three genes in the low-risk and high-risk groups. The CYP2B7P and CACNA2D2 were highly expressed in low-risk group. The expression of KRT6A in low-risk group was lower than that in high-risk group; Table S1: The basic information of the 24 lactate-related genes (LRGs). Table S2: GSVA results between LRG clusters; Table S3: The result of differentially expressed genes (DEGs) statistically significant by univariate Cox analysis.
