# Supplementary material for: Molecular Characterization and Prognosis of Lactate-Related Genes in Lung Adenocarcinoma
Source: Curr Oncol. 2023 Feb 27;30(3):2845–61. doi: 10.3390/curroncol30030217 (PMC10047707; doi:10.3390/curroncol30030217)
Supplement: Supplementary file 1 [file curroncol-30-00217-s001.zip › curroncol-2213382-supplementary.pdf]

Current Oncology

Supplementals

Molecular Characterization and Prognosis of Lactate-Related Genes in Lung Adenocarcinoma

Zixin Guo [1,2,†], Liwen Hu [1,3,†], Qingwen Wang [1,3], Yujin Wang [1,3], Xiao-Ping Liu [4], Chen Chen [2,5], Sheng Li [2,5,*] and Weidong Hu [1,3,*]

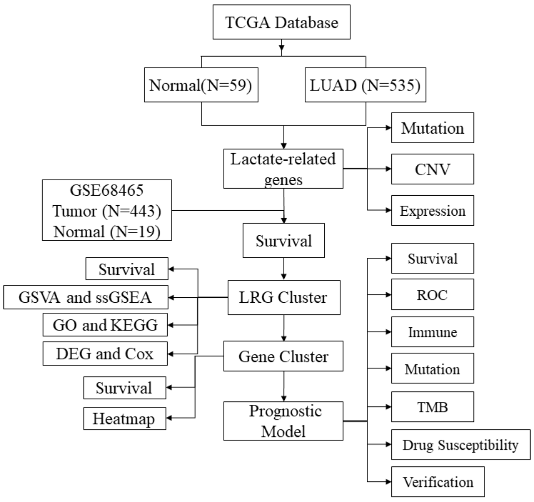

Figure S1. Flow diagram of this study.

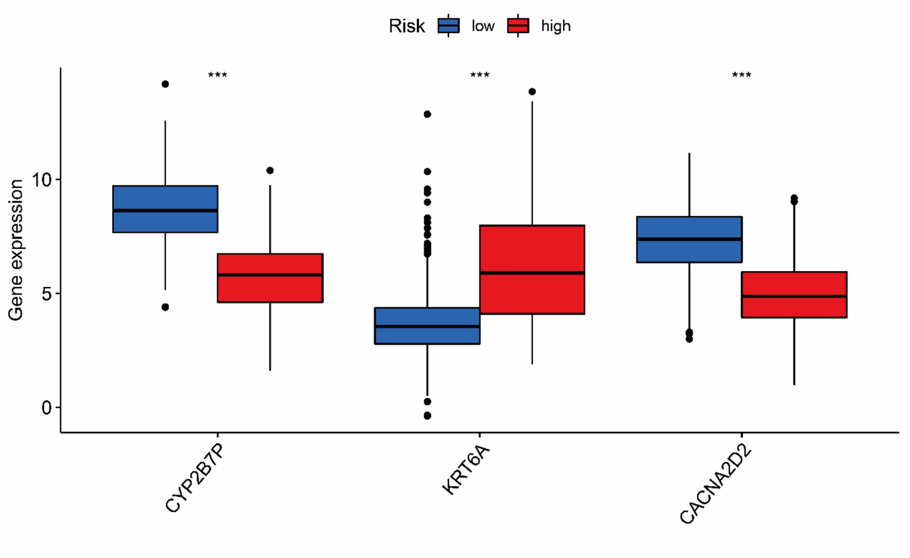

Figure S2. The expression difference of these three genes in the low-rsik and high-risk groups. *** $P < 0.001$.

Table S1. The basic information of the 24 LRGs.

Gene	Type
ACTN3	Lactate
HAGH	Lactate
HIF1A	Lactate
LDHA	Lactate
LDHAL6A	Lactate
LDHAL6B	Lactate
LDHB	Lactate
LDHC	Lactate
LDHD	Lactate
PARK7	Lactate
PER2	Lactate
PFKFB2	Lactate
PNKD	Lactate
SLC16A1	Lactate
SLC16A3	Lactate
SLC16A7	Lactate
SLC16A8	Lactate
SLC25A12	Lactate
SLC5A12	Lactate
SLC5A8	Lactate
TIGAR	Lactate
TP53	Lactate
MYC	Lactate
EMB	Lactate

Table S2. GSVA results between LRGclusters.

id	t	P.Value	adj.P.Val
CELL CYCLE	-20.29878	1.59E-76	2.91E-74
DNA REPLICATION	-16.93948	1.58E-56	1.44E-54
MISMATCH REPAIR	-15.98554	3.45E-51	2.11E-49
P53 SIGNALING PATHWAY	-15.55770	7.51E-49	3.44E-47
OOCYTE MEIOSIS	-15.52779	1.09E-48	3.99E-47
HOMOLOGOUS RECOMBINATION	-15.02247	5.58E-46	1.70E-44
PYRIMIDINE METABOLISM	-13.66510	5.55E-39	1.45E-37
NUCLEOTIDE EXCISION REPAIR	-13.64525	6.97E-39	1.49E-37
PROTEASOME	-13.64108	7.31E-39	1.49E-37
PROGESTERONE MEDIATED OOCYTE MATURATION	-12.48103	3.05E-33	5.58E-32
PATHOGENIC ESCHERICHIA COLI INFECTION	-12.32417	1.65E-32	2.74E-31
DRUG METABOLISM CYTOCHROME P450	12.04380	3.24E-31	4.95E-30
UBIQUITIN MEDIATED PROTEOLYSIS	-11.94019	9.63E-31	1.36E-29
BASE EXCISION REPAIR	-11.90277	1.42E-30	1.86E-29
PRIMARY BILE ACID BIOSYNTHESIS	11.60977	2.96E-29	3.61E-28
SPLICEOSOME	-11.02773	1.03E-26	1.18E-25
RNA DEGRADATION	-11.01505	1.17E-26	1.26E-25
OTHER GLYCAN DEGRADATION	10.76467	1.36E-25	1.38E-24
ALPHA LINOLENIC ACID METABOLISM	10.60403	6.37E-25	6.14E-24
TASTE TRANSDUCTION	10.52318	1.38E-24	1.26E-23
HISTIDINE METABOLISM	10.43113	3.30E-24	2.88E-23
BASAL TRANSCRIPTION FACTORS	-10.42299	3.57E-24	2.97E-23
ALDOSTERONE REGULATED SODIUM REABSORPTION	10.31601	9.76E-24	7.77E-23
SMALL CELL LUNG CANCER	-10.10848	6.72E-23	5.13E-22
LINOLEIC ACID METABOLISM	10.08606	8.27E-23	6.05E-22
FATTY ACID METABOLISM	9.91526	3.94E-22	2.78E-21
GLYCEROPHOSPHOLIPID METABOLISM	9.73710	1.97E-21	1.33E-20
NON HOMOLOGOUS END JOINING	-9.60155	6.58E-21	4.30E-20
PURINE METABOLISM	-9.31591	8.00E-20	5.05E-19
METABOLISM OF XENOBIOTICS BY CYTOCHROME P450	9.25647	1.34E-19	8.15E-19
RETINOL METABOLISM	9.15885	3.08E-19	1.82E-18
ONE CARBON POOL BY FOLATE	-9.00644	1.12E-18	6.40E-18
ARACHIDONIC ACID METABOLISM	8.84562	4.28E-18	2.38E-17
ABC TRANSPORTERS	8.46816	9.23E-17	4.97E-16
RNA POLYMERASE	-8.44129	1.14E-16	5.98E-16
SULFUR METABOLISM	8.43055	1.25E-16	6.33E-16
TYROSINE METABOLISM	8.42610	1.29E-16	6.39E-16
STEROID HORMONE BIOSYNTHESIS	8.12776	1.34E-15	6.44E-15
CALCIUM SIGNALING PATHWAY	7.99939	3.58E-15	1.68E-14
PANCREATIC CANCER	-7.92710	6.19E-15	2.77E-14
NITROGEN METABOLISM	7.92694	6.20E-15	2.77E-14
RENAL CELL CARCINOMA	-7.91715	6.67E-15	2.91E-14
FRUCTOSE AND MANNOSE METABOLISM	-7.81166	1.47E-14	6.27E-14
PEROXISOME	7.47274	1.76E-13	7.33E-13
VALINE LEUCINE AND ISOLEUCINE DEGRADATION	7.46643	1.84E-13	7.50E-13
PENTOSE PHOSPHATE PATHWAY	-7.45048	2.07E-13	8.23E-13
VASCULAR SMOOTH MUSCLE CONTRACTION	7.28569	6.66E-13	2.59E-12
NOD LIKE RECEPTOR SIGNALING PATHWAY	-7.18804	1.32E-12	5.03E-12
CYSTEINE AND METHIONINE METABOLISM	-7.18439	1.35E-12	5.05E-12

GALACTOSE METABOLISM	-7.16484	1.55E-12	5.67E-12
CIRCADIAN RHYTHM MAMMAL	7.13654	1.88E-12	6.76E-12
PROXIMAL TUBULE BICARBONATE RECLAMATION	7.07685	2.84E-12	9.99E-12
PPAR SIGNALING PATHWAY	7.02460	4.06E-12	1.40E-11
MELANOGENESIS	7.00554	4.62E-12	1.57E-11
VASOPRESSIN REGULATED WATER REABSORPTION	6.98687	5.24E-12	1.74E-11
GLYOXYLATE AND DICARBOXYLATE METABOLISM	-6.97761	5.58E-12	1.82E-11
GLYCOLYSIS GLUCONEOGENESIS	-6.89338	9.85E-12	3.16E-11
GLYCOSAMINOGLYCAN BIOSYNTHESIS KERATAN SULFATE	-6.77916	2.11E-11	6.64E-11
GNRH SIGNALING PATHWAY	6.59510	7.00E-11	2.17E-10
BASAL CELL CARCINOMA	6.48183	1.45E-10	4.41E-10
BUTANOATE METABOLISM	6.39812	2.45E-10	7.36E-10
BLADDER CANCER	-6.33740	3.59E-10	1.06E-09
CHRONIC MYELOID LEUKEMIA	-6.15099	1.13E-09	3.28E-09
TRYPTOPHAN METABOLISM	5.98928	2.97E-09	8.50E-09
NEUROACTIVE LIGAND RECEPTOR INTERACTION	5.94266	3.92E-09	1.10E-08
GLYCOSAMINOGLYCAN DEGRADATION	5.88572	5.47E-09	1.52E-08
RIBOFLAVIN METABOLISM	-5.83110	7.51E-09	2.05E-08
STARCH AND SUCROSE METABOLISM	-5.67960	1.79E-08	4.81E-08
PHOSPHATIDYLINOSITOL SIGNALING SYSTEM	5.54102	3.88E-08	1.03E-07
TYPE II DIABETES MELLITUS	5.48639	5.25E-08	1.37E-07
LYSOSOME	5.46182	6.00E-08	1.55E-07
GLYCOSYLPHOSPHATIDYLINOSITOL GPI ANCHOR BIOSYNTHESIS	5.43318	7.02E-08	1.78E-07
CITRATE CYCLE TCA CYCLE	-5.35898	1.05E-07	2.63E-07
PROTEIN EXPORT	-5.31315	1.34E-07	3.31E-07
GLYCEROLIPID METABOLISM	5.26069	1.77E-07	4.32E-07
COMPLEMENT AND COAGULATION CASCADES	5.13260	3.46E-07	8.32E-07
PATHWAYS IN CANCER	-5.12416	3.61E-07	8.58E-07
AMINOACYL TRNA BIOSYNTHESIS	-5.07927	4.55E-07	1.07E-06
ADIPOCYTOKINE SIGNALING PATHWAY	4.94381	9.04E-07	2.09E-06
LONG TERM DEPRESSION	4.92460	9.95E-07	2.28E-06
GLYCOSPHINGOLIPID BIOSYNTHESIS GANGLIO SERIES	4.87303	1.28E-06	2.90E-06
HEDGEHOG SIGNALING PATHWAY	4.82000	1.67E-06	3.72E-06
CYTOSOLIC DNA SENSING PATHWAY	-4.80244	1.82E-06	4.01E-06
ASCORBATE AND ALDARATE METABOLISM	4.65030	3.78E-06	8.23E-06
FC GAMMA R MEDIATED PHAGOCYTOSIS	-4.56314	5.69E-06	1.23E-05
GAP JUNCTION	-4.42373	1.08E-05	2.30E-05
PARKINSONS DISEASE	-4.41928	1.10E-05	2.32E-05
INOSITOL PHOSPHATE METABOLISM	4.35594	1.47E-05	3.05E-05
PYRUVATE METABOLISM	-4.29727	1.90E-05	3.92E-05
ASTHMA	4.26591	2.19E-05	4.45E-05
MATURITY ONSET DIABETES OF THE YOUNG	4.17589	3.24E-05	6.51E-05
HUNTINGTONS DISEASE	-4.14641	3.68E-05	7.31E-05
OLFACTORY TRANSDUCTION	4.11829	4.15E-05	8.16E-05
DILATED CARDIOMYOPATHY	4.05416	5.44E-05	0.00010587
REGULATION OF ACTIN CYTOSKELETON	-4.00887	6.57E-05	0.00012662
ALZHEIMERS DISEASE	-3.98104	7.38E-05	0.00014064
CELL ADHESION MOLECULES CAMS	3.86833	0.000117	0.00022065
COLORECTAL CANCER	-3.72576	0.000206	0.0003848
WNT SIGNALING PATHWAY	3.69723	0.00023	0.00042568
LONG TERM POTENTIATION	3.69430	0.000233	0.00042625

TIGHT JUNCTION	3.63052	0.000298	0.00053951
BETA ALANINE METABOLISM	3.60706	0.000326	0.0005842
PORPHYRIN AND CHLOROPHYLL METABOLISM	3.58149	0.000359	0.00063737
NICOTINATE AND NICOTINAMIDE METABOLISM	-3.55008	0.000404	0.00071046
TOLL LIKE RECEPTOR SIGNALING PATHWAY	-3.45737	0.000569	0.0009923
CARDIAC MUSCLE CONTRACTION	3.45360	0.000577	0.00099662
GLIOMA	-3.43535	0.000617	0.00105539
GLYCINE SERINE AND THREONINE METABOLISM	3.28976	0.001039	0.00176077
PHENYLALANINE METABOLISM	3.25957	0.001155	0.00193901
MAPK SIGNALING PATHWAY	3.22490	0.001303	0.00216709
FOCAL ADHESION	-3.21654	0.001341	0.00221048
INSULIN SIGNALING PATHWAY	3.20713	0.001385	0.00226288
GLYCOSAMINOGLYCAN BIOSYNTHESIS CHONDROITIN SULFATE	-3.18130	0.001513	0.00245045
PROPANOATE METABOLISM	3.17621	0.00154	0.00247153
FOLATE BIOSYNTHESIS	-3.16665	0.001591	0.00253113
RENIN ANGIOTENSIN SYSTEM	3.10905	0.001932	0.003048
AMINO SUGAR AND NUCLEOTIDE SUGAR METABOLISM	-3.04201	0.002414	0.00377548
ETHER LIPID METABOLISM	3.00453	0.002729	0.004232
CHEMOKINE SIGNALING PATHWAY	-2.96988	0.003053	0.00469519
THYROID CANCER	-2.95964	0.003156	0.00481219
AMYOTROPHIC LATERAL SCLEROSIS ALS	-2.93256	0.003442	0.00520498
ARGININE AND PROLINE METABOLISM	2.89526	0.003874	0.00581121
REGULATION OF AUTOPHAGY	-2.87343	0.00415	0.00617393
HYPERTROPHIC CARDIOMYOPATHY HCM	2.80828	0.005082	0.00749937
N GLYCAN BIOSYNTHESIS	-2.80082	0.005199	0.00761206
GLYCOSPHINGOLIPID BIOSYNTHESIS LACTO AND NEOLACTO SERIES	2.77147	0.005688	0.0082612
VIBRIO CHOLERAE INFECTION	2.76090	0.005874	0.00845526
STEROID BIOSYNTHESIS	2.75866	0.005914	0.00845526
MTOR SIGNALING PATHWAY	-2.65029	0.008174	0.01159616
ECM RECEPTOR INTERACTION	-2.57776	0.010092	0.01420677
AUTOIMMUNE THYROID DISEASE	2.50320	0.012472	0.01742314
PRION DISEASES	-2.47260	0.013585	0.01883438
VIRAL MYOCARDITIS	2.37635	0.01768	0.02432649
FC EPSILON RI SIGNALING PATHWAY	2.34828	0.019062	0.02603232
JAK STAT SIGNALING PATHWAY	2.34119	0.019426	0.02633243
RIBOSOME	-2.33401	0.0198	0.02664326
GLUTATHIONE METABOLISM	-2.32800	0.020119	0.02687399

Table S3. The result of DEGs statistically significant by univariate Cox analysis.

id	HR	HR.95L	HR.95H	p-value
SLC16A1	1.2154859	1.1252859	1.3129162	7.04E-07
SELENBP1	0.860448	0.8033592	0.9215938	1.78E-05
CACNA2D2	0.8866461	0.8419193	0.933749	5.23E-06
FOLR1	0.925208	0.8813828	0.9712124	0.0016909
CRYM	0.8818073	0.8316102	0.9350343	2.60E-05
CYP2B7P	0.8892904	0.8502847	0.9300855	2.94E-07
CYP4B1	0.9030212	0.8677263	0.9397517	5.31E-07
GGTLC1	0.868945	0.8189198	0.9220261	3.43E-06
MMP12	1.0676001	1.0161574	1.1216471	0.0094293
SFTPB	0.8951319	0.8618501	0.9296989	1.00E-08
NKX2-1	0.877502	0.835529	0.9215835	1.74E-07
PGC	0.9439946	0.914622	0.9743105	0.000352
PIGR	0.9068739	0.8677094	0.947806	1.43E-05
C1orf116	0.9273928	0.885454	0.971318	0.0014105
SERPINB5	1.12931	1.0841955	1.1763018	5.03E-09
C4BPA	0.922769	0.8824678	0.9649107	0.0004192
SFTPD	0.9245508	0.8899903	0.9604535	5.44E-05
WIF1	0.9532599	0.9175319	0.9903793	0.0140505
KRT6A	1.1186796	1.0802926	1.1584306	3.07E-10
SFTPC	0.9569323	0.9307219	0.9838809	0.0018912
SCGB1A1	0.9600958	0.9323586	0.9886581	0.0064773
